# Co-infection of dengue and Zika viruses mutually enhances viral replication in the mosquito *Aedes aegypti*

**DOI:** 10.1186/s13071-023-05778-1

**Published:** 2023-05-11

**Authors:** Daniel Chieh-Ding Lin, Shih-Che Weng, Po-Nien Tsao, Justin Jang Hann Chu, Shin-Hong Shiao

**Affiliations:** 1 Department of Biochemical Science and Technology, College of Life Science, National Taiwan University, Taipei, Taiwan; 2 Department of Tropical Medicine and Parasitology, College of Medicine, National Taiwan University, Taipei, Taiwan; 3 Department of Pediatrics, National Taiwan University Hospital, Taipei, Taiwan; 4 Research Center for Developmental Biology & Regenerative Medicine, National Taiwan University, Taipei, Taiwan; 5 Laboratory of Molecular RNA Virology and Antiviral Strategies, Department of Microbiology and Immunology, Yong Loo Lin School of Medicine, National University of Singapore, Singapore, Singapore

**Keywords:** *Aedes aegypti*, Dengue virus, Zika virus, Co-infection, NS5 protein

## Abstract

**Background:**

The mosquito *Aedes aegypti* transmits two of the most serious mosquito-borne viruses, dengue virus (DENV) and Zika virus (ZIKV), which results in significant human morbidity and mortality worldwide. The quickly shifting landscapes of DENV and ZIKV endemicity worldwide raise concerns that their co-circulation through the *Ae. aegypti* mosquito vector could greatly exacerbate the disease burden in humans. Recent reports have indicated an increase in the number of co-infection cases in expanding co-endemic regions; however, the impact of co-infection on viral infection and the detailed molecular mechanisms remain to be defined.

**Methods:**

C6/36 (*Aedes albopictus*) cells were cultured in Dulbecco's modified Eagle medium/Mitsuhashi and Maramorosch Insect Medium (DMEM/MM) (1:1) containing 2% heat-inactivated fetal bovine serum and 1× penicillin/streptomycin solution. For virus propagation, the cells were infected with either DENV serotype 2 (DENV2) strain 16681 or ZIKV isolate Thailand/1610acTw (MF692778.1). Mosquitoes (*Ae. aegypti* UGAL [University of Georgia Laboratory]/Rockefeller strain) were orally infected with DENV2 and ZIKV through infectious blood-feeding.

**Results:**

We first examined viral replication activity in cells infected simultaneously, or sequentially, with DENV and ZIKV, and found interspecies binding of viral genomic transcripts to the non-structural protein 5 (NS5). When we challenged *Ae. aegypti* mosquitos with both DENV2 and ZIKV sequentially to probe similar interactions, virus production and vector susceptibility to infection were significantly enhanced.

**Conclusions:**

Our results suggest that DENV2 and ZIKV simultaneously establishing infection in the *Ae. aegypti* mosquito vector may augment one another during replication. The data also implicate the homologous NS5 protein as a key intersection between the flaviviruses in co-infection, highlighting it as a potential target for vector control.

**Supplementary Information:**

The online version contains supplementary material available at 10.1186/s13071-023-05778-1.

## Background

Mosquito-borne diseases are one of the most significant public health burdens [1–5]. Human activities, urbanization, and climate change are increasingly bringing more human hosts in contact with disease vectors [2, 6–9]. Currently, half of the global population is at risk for dengue virus (DENV) infection [2, 8, 10]. In the aftermath of the 2015–2016 Zika virus (ZIKV) outbreak, which exposed more than 130 million people to infection, the virus remains endemic to tropical and subtropical regions [11].

Sharing a common vector in *Aedes aegypti*, DENV and ZIKV endemicity may expand in concert, resulting in widespread co-circulation [12, 13]. Thus, DENV-ZIKV synergy presents a bleak outlook for the near future with a growing proportion of the global population living under threat of simultaneous infection by both flaviviruses. As DENV-ZIKV co-circulation expands worldwide to affect currently low-risk or virus-free regions, mosquito vectors will have increased opportunities to receive and transmit both viruses [5]. Recent reports of DENV-ZIKV co-infection corroborate this prediction and reflect a proliferating synergy that has been overlooked. This may be the result of systemic underreporting engendered by difficulties in the differential diagnosis and detection of asymptomatic infections [14–16].

One cross-sectional study of the ZIKV epidemic in Colombia detected 8.8% of the DENV-ZIKV serotype among 34 co-infection cases [15]. Another study in southern Mexico randomly sampled a cohort of pregnant women during a non-epidemic period and found a relatively high proportion (2%) with DENV-ZIKV co-infection [17]. A previous report revealed that DENV infections occurred during the same period, highlighting the concerning extent of silent transmission of ZIKV with DENV [17, 18]. Besides underreporting, underlying this phenomenon may be antibody-dependent enhancement (ADE) between DENV and ZIKV in co-endemic areas. Studies support ADE of ZIKV infection by anti-DENV antibodies [4, 19–21], which not only may increase disease severity, but also may drive ZIKV transmission into primarily DENV-endemic areas. Similar results have been suggested for DENV, in which ZIKV-mediated ADE increases the propensity for severe disease and enables DENV to persist and proliferate in primarily ZIKV-endemic regions [22, 23]. These trends are extremely troubling as they suggest a mutualistic relationship between DENV and ZIKV co-circulating in the *Ae. aegypti* urban transmission cycle, which could lead to further expansion.

In a mosquito vector simultaneously housing both DENV and ZIKV, either from a single co-infected patient or from separate infectious BMs, the confluence of viral replication and antiviral suppression pathways may produce distinct vector competence phenotypes, possibly to affect enhanced susceptibility and transmissibility. Thus, it is important to clarify DENV-ZIKV co-infection in the mosquito and determine the underlying molecular interactions for the development of effective vector control strategies. To date, only limited studies of arbovirus co-infection have been reported, which demonstrate the susceptibility of *Ae. aegypti* to DENV-ZIKV co-infection and supported the prospect of transmitting both viruses simultaneously [12, 24]. The specific effect of DENV-ZIKV co-infection on viral replication, however, remains to be elucidated.

In this study, we determined the effects of DENV and ZIKV co-infection on viral replication in *Ae. aegypti* to identify the specific molecular interactions involved. We first examined viral replication dynamics in cells infected simultaneously or sequentially with DENV and ZIKV. We report interspecies binding of viral genomic transcripts to the non-structural protein 5 (NS5). We then challenged *Ae. aegypti* mosquitos with both DENV serotype 2 (DENV2) and ZIKV sequentially to identify similar interactions, and found that virus production and vector susceptibility to infection were significantly enhanced. Our results suggest that DENV2 and ZIKV simultaneously establish infection in the *Ae. aegypti* vector, which may mutually augment one another during replication. The data also implicate the homologous NS5 protein as a key intersection between the flaviviruses in co-infection, highlighting it as a potential target for vector control.

## Methods

### Mosquitos

*Aedes aegypti* mosquitos (UGAL [University of Georgia Laboratory]/Rockefeller strain) were maintained at 28 °C and 70% relative humidity under a photoperiod of 12:12 h as described previously [25, 26]. Hatched larvae were transferred to plastic containers filled with water and fed daily with yeast extract. Pupae were collected and transferred to an insect dorm where emerging mosquitos were fed using cotton balls soaked in 10% sucrose solution. Female mosquitos 3–5 days post-eclosion (PE) were used for the experiments, and the sucrose-soaked cotton balls were removed at least 12 h before blood-feeding. Female mosquitos were permitted to feed on an anesthetized Institute of Cancer Research (ICR) strain mouse for 15–30 min. ICR strain mice were anesthetized via intraperitoneal injection of Avertin at a dose of 0.2 ml/10 g body weight. All animal procedures and experimental protocols were approved by the institutional Association for Assessment and Accreditation of Laboratory Animal Care (AAALAC) International-accredited facility and the Committee on the Ethics of Animal Experiments at the National Taiwan University College of Medicine (IACUC Approval No: 20200210).

### Cell culture and viruses

C6/36 (*Ae. albopictus*) cells were cultured in Dulbecco's modified Eagle medium/Mitsuhashi and Maramorosch Insect Medium (DMEM/MM) (1:1) containing 2% heat-inactivated fetal bovine serum and 1× penicillin/streptomycin solution. For virus propagation, the cells were infected with either DENV2 strain 16681 or ZIKV isolate Thailand/1610acTw (MF692778.1) at 0.01 multiplicity of infection (MOI). The culture supernatant was harvested 7 days post-infection (dpi) and stored at −80 °C. To quantify the viral titer, the supernatant was subjected to examination by plaque assay as described previously [27]. Approximately 1.0 × 10^7^ plaque-forming units (PFU)/ml of DENV2 and ZIKV were used to infect the mosquitos.

### Immunofluorescence assay (IFA)

*Aedes albopictus* C6/36 cells were dispensed onto a cover glass and cultured in 12-well plates overnight. The virus suspension (MOI = 1 or 10) was then added to each well. Following virus adsorption at 28 °C for 2 h, the suspension was removed and replaced with fresh medium. At 2 dpi, the cover glass was fixed in 4% paraformaldehyde (Electron Microscopy Sciences, Hatfield, PA, USA) for 30 min. The fixative was removed and the cover glass was rinsed in phosphate-buffered saline (PBS), and incubated for 1 h in 0.1% Triton X-100 in PBS for cell permeabilization, and blocked with blocking buffer (1% bovine serum albumin [BSA], 0.5% Triton X-100 in PBS) for 1 h. Monoclonal mouse anti-non-structural protein 1 (NS1) antibody (YH0023) (Yao-Hong Biotechnology Inc., Taipei, Taiwan) and ZIKV-specific envelope protein antibody (GTX133314) were used as the primary antibody (1:1000) to detect DENV and ZIKV antigens in the cells. Cells were then incubated with a 1:500 dilution of goat anti-mouse antibody conjugated to Alexa Fluor 488 fluorochrome (Molecular Probes Inc., Eugene, OR, USA). Finally, the cover glass was mounted with a DAPI-containing medium for confocal microscopy (ZEISS, LSM 510 META confocal microscope).

### RNA extraction and reverse transcription

C6/36 cell pellets or homogenized individual mosquitos were collected in 1.5 ml tubes containing 0.5 ml TRIzol Reagent (Invitrogen). Samples were homogenized with a rotor–stator homogenizer and centrifuged at 13,000 rpm for 10 min at 4 °C. The supernatants were then transferred to new tubes each containing 0.1 ml chloroform (J.T.Baker) and mixed thoroughly. After 3 min of incubation on ice, samples were then centrifuged at 13,000 rpm for 15 min at 4 °C, and the supernatants were transferred to new tubes containing 0.25 ml isopropanol (J.T.Baker). Samples were gently mixed and stored at −80 °C for 30 min. After precipitation, the samples were once again centrifuged at 13,000 rpm for 30 min at 4 °C. The supernatants were discarded and the RNA pellets were washed with 0.5 ml 75% ethanol (Taiwan Burnett International Co., Ltd.). The samples were then centrifuged at 8000 rpm for 5 min at 4 °C and the supernatants were discarded. Finally, the RNA pellets were dried in a laminar flow cabinet and dissolved in DEPC-H_2_O. After Baseline-ZERO™ DNase (Epicentre) treatment, purified RNA samples were stored at −80 °C. The RNA concentrations were quantified using an ultraviolet–visible (UV–Vis) spectrophotometer (NanoDrop 2000, Thermo Fisher) and diluted with DEPC-H_2_O to a concentration of 1 μg/μl. The RNA (1 μg/μl) was then reverse-transcribed to complementary DNA (cDNA) using the High-Capacity cDNA Reverse Transcription Kit (Applied Biosystems) and stored at −20 °C.

### Quantitative real-time polymerase chain reaction (qRT-PCR)

Quantitative RT-PCR quantification was performed using SYBR Green chemistry. The cDNA samples were quantified with the KAPA SYBR FAST Universal qPCR kit (KAPA). PCR consisted of an initial denaturation at 95 °C for 3 min, then 40 cycles at 94 °C for 3 s each, followed by 40 s at 60 °C. Fluorescence readings were measured at 72 °C after each cycle. The target gene signal was detected and analyzed with the ABI 7900HT Fast Real-Time PCR System and relative quantification results were normalized to the expression of the ribosomal protein S7 gene as an internal control [28–30].

### Plaque assay

Whole bodies of individual mosquitos were collected in 100 μl of serum-free medium and stored at −80 °C. BHK-21 cells were seeded in a 24-well tissue culture plate and incubated at 37 °C overnight. Homogenized suspensions of individual whole bodies were centrifuged at 18,928×*g* for 30 min and kept on ice. Cell monolayers were rinsed with PBS and incubated with 200 μl of 10-fold serial diluted homogenized mosquito suspensions for 2 h. Following viral adsorption, 500 μl of 1% methylcellulose cell medium was added to each well and the culture plates were kept in an incubator at 28 °C for 5 days. The plates were then fixed at room temperature with 4% formaldehyde for 1 h and stained with 1% crystal violet for 30 min. Plaques were then quantified manually [27].

### Cross-linking and immunoprecipitation followed by reverse transcription and PCR (CLIP-PCR)

C6/36 cells were seeded in a T75 flask and incubated at 28 °C overnight, then the virus suspension was added. Following adsorption at 28 °C for 2 h, the virus suspension was removed and replaced with fresh medium. At 2 dpi, the cells were fixed in 1% paraformaldehyde (Electron Microscopy, Hatfield, PA, USA) for 30 min. The fixative was removed and cells were resuspended in 1 ml of protein lysis buffer (50 mM Tris, pH 7.4, 1% IGEPAL, 0.25% sodium deoxycholate, 150 mM NaCl, 1 mM ethylenediaminetetraacetic acid (EDTA), 1 mM phenylmethyl-sulfonylfluoride, 1× protease inhibitor mixture, and 1× phosphatase inhibitor mixture), then homogenized using a rotor–stator homogenizer. The samples were transferred to a QIAshredder™ column (Qiagen) for solubilization of cross-linked complexes. The eluted samples were collected and transferred to new Eppendorf tubes at −80 °C. Protein G-agarose beads (20 μl, packed volume) were coated with a specific antibody for 2 h at 4 °C followed by extensive washing with RIPA buffer containing protease inhibitors. The cell lysate (500 μl) was diluted with RIPA buffer (500 μl), mixed with the antibody-coated beads, and incubated with rotation for 4 h at 4 °C. The beads were collected using a mini-centrifuge at 700×*g* for 5 min at 4 °C and the supernatant was removed. The antibody-coupled beads were washed three times by adding 1 ml of RIPA buffer and centrifuging at 700×*g* and 4 °C for 5 min. The beads containing the immunoprecipitated samples were collected, resuspended in 50 μl of TE buffer, and incubated at 70 °C for 45 min to reverse the cross-links. The RNA was extracted from these samples using TRIzol Reagent according to the manufacturer’s protocol (Invitrogen). RNA (1 μg/μl) was then reverse-transcribed to cDNA using the High-Capacity cDNA Reverse Transcription Kit (Applied Biosystems) and stored at −20 °C.

### Oral infection of virus

Female mosquitoes were infected by feeding them with an infectious blood meal prepared by mixing 200 μl of mouse whole blood, 50 μl of 1 mM ATP, and 250 μl of DENV2 16681 strain (2.5 × 10^6^ PFU in 250 μl) using folded Parafilm-M. Prior to the blood meal, the mosquitoes were starved through sugar deprivation for 12 h. After feeding, each mosquito was observed under a stereomicroscope to confirm successful blood intake. The mosquitoes were then kept at 28 °C and 70% relative humidity with a 12 h:12 h light–dark cycle, as described previously [31, 32]. Mosquitos were captured 12 h ahead of day 0 and presented with blood meal (BM), DENV2, ZIKV, or maintained on sugar feeding. On day 5, one group was given a second BM (BM-BM), and others were challenged with DENV2 or ZIKV. At 7 dpi (day 12), whole mosquito bodies were collected and homogenized. Relative viral genome expression of DENV2 and ZIKV was determined by qRT-PCR normalized to the ribosomal *S7* protein gene. Infectious viral titers were quantified via plaque assay as described above. Each of the six oral challenge schemes consisted of at least five biologically independent cohorts.

### Statistical analysis

All statistical analyses were performed using GraphPad Prism 8 software. One-way analysis of variance (ANOVA) or the Kruskal–Wallis test by ranks was used to compare independent cohorts in each set of experiments. Post hoc analyses were performed for variance tests bearing significance at *α* = 0.05 using Tukey’s and Dunn’s multiple comparisons tests, respectively.

### Graphical illustrations

The graphical abstract and parts of Figs. 2 and 4 were made with Biorender.com. A publication license was obtained for each figure. DENV and ZIKV particles used in the graphical abstract were provided courtesy of David Goodsell (Scripps Research, CA, USA), made publicly available at PDB-101 under a CC BY 4.0 license.

## Results

### Simultaneous infection with DENV2 and ZIKV modulates viral NS1 subcellular localization and viral replication in mosquito cells

**Fig. 1:**
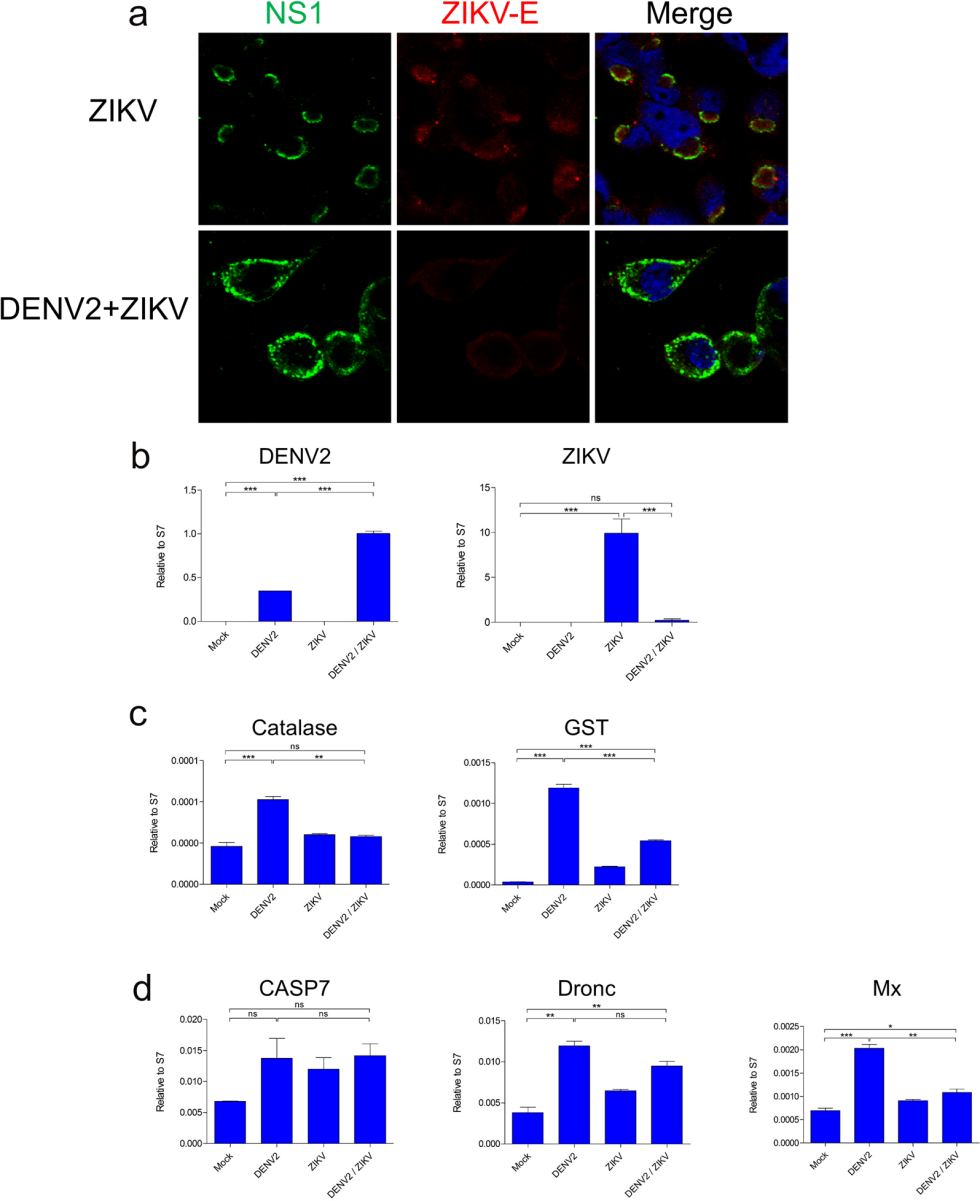
Replication, subcellular localization, and systematic responses following simultaneous DENV2/ZIKV co-infection of C6/36 mosquito cells. **A** Immunofluorescence of the flaviviral NS1 (green) in ZIKV single-infected and DENV2/ZIKV co-infected cells, with DAPI (blue)-stained DNA demarcating nuclei. Scale bars, 10 μm. **B** Relative levels of DENV and ZIKV viral genomes in cell lysate at 2 days post-infection (dpi). **C** Relative expression of genes encoding catalase and glutathione S-transferase family proteins in cell lysate at 2 dpi. These results are representative of at least three independent experiments. **D** Relative expression of *casp7*, *dronc*, and *Mx* in cell lysate at 2 dpi. These results are representative of at least three independent experiments. Differences between groups were demonstrated to be statistically significant using Tukey’s multiple comparisons test; *P < 0.05, **P < 0.01, ***P < 0.001

To investigate DENV-ZIKV co-infection in the mosquito, we infected mosquito cells simultaneously with DENV2 and ZIKV, infecting mosquito C6/36 cells with ZIKV (single infection) and DENV2/ZIKV (co-infection) at an MOI of 10. At 2 dpi, the subcellular localization of viral NS1 protein was characterized via IFA using anti-NS1 antibody (green). Remarkably, simultaneous co-infection (DENV/ZIKV) modulated the subcellular localization of viral NS1 produced by both viruses (Fig. 1A). Departing from our observations in ZIKV single-infected cells, wherein NS1 associated into vesicle-like structures, viral NS1 in DENV2/ZIKV co-infected cells localized in the cytoplasm, as in DENV2 single-infected cells (Additional file 1: Fig. S1a). In addition, the rate of cellular mortality was observed to be greater in cells infected with DENV2 than in those infected with ZIKV (Additional file 1: Fig. S1b). Granted, NS1 detection may not directly correspond to changes in viral genomic replication and localization in vitro.

We then quantified the effects of simultaneous DENV2/ZIKV co-infection on viral genomic replication (Fig. 1b). Our results show that, compared with DENV2 single-infected cells, DENV2 viral genome replication in DENV2/ZIKV co-infected cells was significantly higher at equal MOI. Conversely, cellular ZIKV genome replication was drastically suppressed in co-infected cells. Fluorescence imaging corroborates this finding: ZIKV envelope (E) protein was hardly present in DENV2/ZIKV co-infected cells; in ZIKV-infected cells, ZIKV-E was not only present at NS1-localized sites but was also dispersed throughout the cytoplasm (Fig. 1a). Viral E protein serves as an indication of replication activity because it associates with the endoplasmic reticulum membrane at the site of ribonucleocapsid assembly, eventually budding off with prM proteins to line the casing of live viral particles.

In order to gain insight into the underlying mechanisms responsible for the differing viral replication rates and levels of cellular mortality observed in cells infected with DENV2 versus ZIKV, we measured the expression levels of genes associated with stress and apoptosis. We found that transcript abundance of catalase and glutathione-S-transferase (GST), enzymes mediating oxidative stress responses to viral infection in *Ae. aegypti*, were significantly downregulated in co-infected cells compared with DENV2 single-infected cells (Fig. 1C). Meanwhile, expression of apoptosis-promoting genes *casp7* and *dronc* did not differ significantly between co-infected and DENV-infected cells, with a significant decrease in *Mx* (Fig. 1D). Overall, we find that co-infection produces a distinct antiviral response profile in terms of oxidative stress, but not apoptotic processes.

Our results demonstrate that, surprisingly, simultaneous DENV2-ZIKV co-infection significantly modulates viral NS1 subcellular localization and viral genomic replication, apparently benefiting DENV2. The fact that viral NS1 co-localizes in co-infected cells raises the interesting possibility that DENV and ZIKV replication may overlap. NS1 proteins serve as scaffolding protein dimers for the flaviviruses’ highly homologous replication complexes (RCs). DENV-ZIKV co-infection presents abundant opportunity for spatio-temporal coordination of viral propagation, with significant implications for viral replication and virus assembly. The divergent outcomes of viral genomic replication observed (DENV2 enhancement, ZIKV attenuation) following co-infection hint at competitive interactions during infection and replication. It is not clear, however, whether the stark contrast between DENV and ZIKV replication in this co-infection model is due to superior engagement of DENV2-NS1, with the cell membrane outcompeting ZIKV-NS1 (Additional file 1: Fig. S1B), or because genomic DENV2 made more efficient use of both DENV2 and ZIKV RCs. To probe this distinction, we employed a sequential co-infection model, which allowed for temporal segregation of DENV2 and ZIKV replication at the level of RC establishment and early genomic replication.

### Sequential infection with DENV and ZIKV similarly modulates viral NS1 subcellular localization and viral replication in mosquito cells

**Fig. 2:**
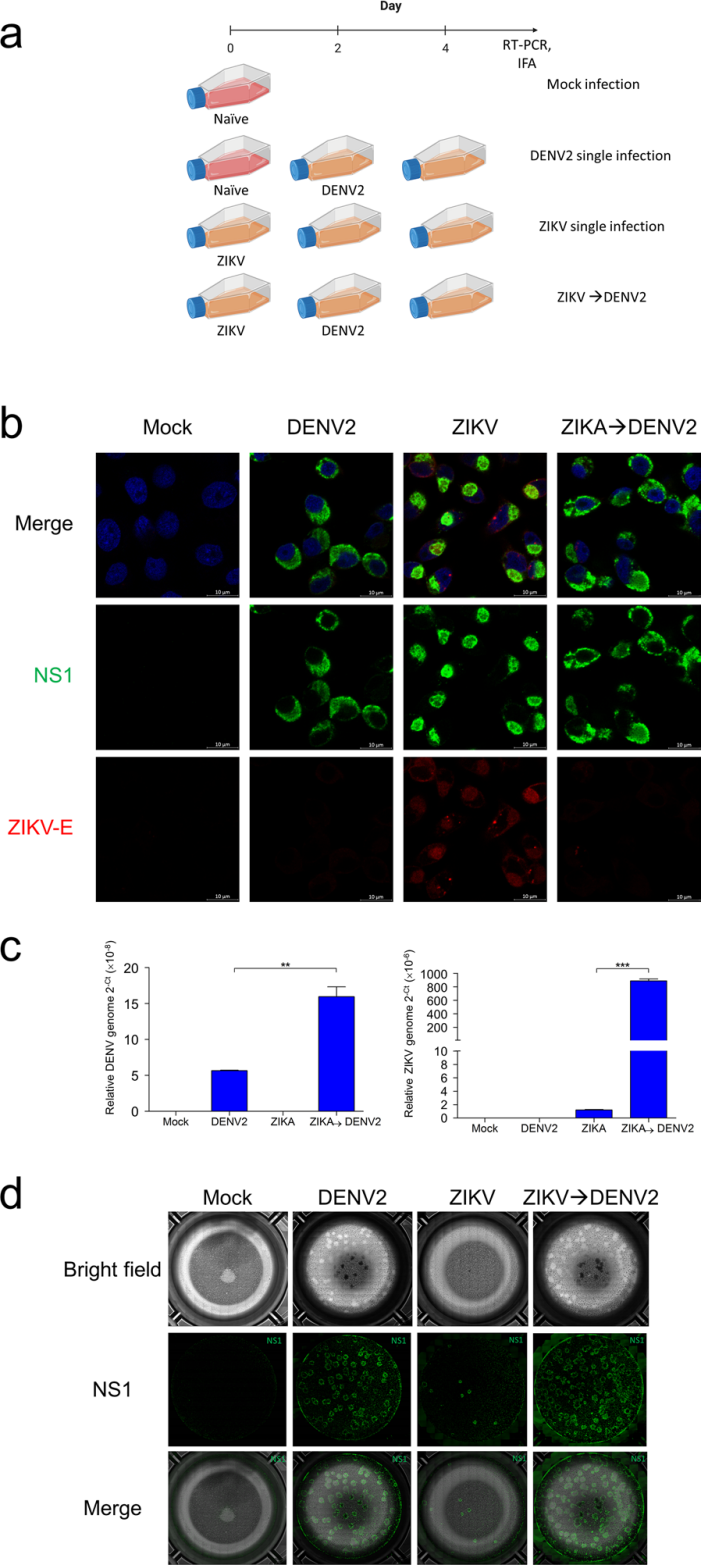
Sequential co-infection modulates viral NS1 subcellular localization and viral replication in mosquito cells. **A** Time course of sequential infection with ZIKV and DENV2.** B** Subcellular localization of flaviviral NS1 (green) and ZIKV-E protein (red) in single-infected and ZIKV → DENV2 sequentially co-infected cells at 2 dpi, with DAPI (blue)-stained DNA demarcating nuclei. Scale bars, 10 μm. **C** Relative levels (2^−(dCt)^) of DENV and ZIKV viral genomes in secreted viruses in the supernatant of cell culture medium at 2 dpi. These results are representative of at least three independent experiments. **D** At 2 dpi, culture supernatants from single-infected and ZIKV → DENV2 sequentially co-infected cells were collected and used in a focus-forming assay. NS1 (green) stained for replicating virus

To evaluate whether the increase in DENV2 replication efficiency observed in simultaneously co-infected cells resulted from more extensive DENV2 RC establishment during initial infection, we inoculated C6/36 cells first with ZIKV, and then with DENV2 (Fig. 2A). This model of sequential co-infection provides ZIKV with the opportunity to establish infection prior to DENV2 inoculation. Consistent with earlier observations (Fig. 1A), viral NS1 in ZIKV-infected cells tended to associate into vesicle-like subcellular structures, but dispersed throughout the cytoplasm in DENV-infected cells (Fig. 2B). As for cells co-infected sequentially with ZIKV, then DENV2, significant overlap of viral NS1 was observed, with some retention of vesicle-like formations present in ZIKV single-infected cells (Fig. 2B). This presentation is consistent with that for simultaneously co-infected cells, and clearly demonstrates that DENV2-ZIKV co-infection in the mosquito cell, regardless of inoculation sequence, modulates viral NS1 localization to allow for possible extensive overlap of the flaviviruses’ replicative activities.

Assessment of viral RNA extracted from secreted viruses in supernatant of these ZIKV → DENV2 sequentially co-infected cells revealed that both DENV2 and ZIKV replication were significantly enhanced (Fig. 2C). ZIKV replication benefited from a spatio-temporal advantage, allowing it to establish RC and initiate replication first. The fact that DENV2 replication also benefited rules out the possibility that superior membrane integration of DENV2-NS1 and its associated RC allowed it to outcompete ZIKV in the simultaneous co-infection model. Instead, an intriguing prospect arises: interspecies interactions may be at work, whereby replicating viruses engage the complementary RC to its benefit.

Before probing further possible interspecies DENV-ZIKV interactions during co-infection, we sought to confirm that our model indeed permitted infectious virion production downstream of viral genomic replication. We inoculated uninfected C6/36 cells with supernatant containing viral particles produced by ZIKV → DENV2 sequentially co-infected cells, staining for viral NS1 to confirm infection and replication initiation (Fig. 2D). The extent to which secreted viruses in co-infected supernatant established infection was clearly superior to DENV2 and ZIKV alone, particularly for ZIKV.

Evidently, DENV2/ZIKV co-infection of mosquito cells modulates NS1 cellular localization and significantly enhances virus production. The use of the sequential infection model provides a high level of spatio-temporal resolution that enables us to identify that the observed effects are a result of the viral replication process. The overlap of DENV2-ZIKV replication loci in co-infected cells likely provides for cross-species interactions resulting in mutual enhancement of replication. Accordingly, we probed directly interspecies molecular interactions during DENV-ZIKV co-infection.

### The replicating DENV2 genome interacts with the ZIKV-NS5 protein

**Fig. 3:**
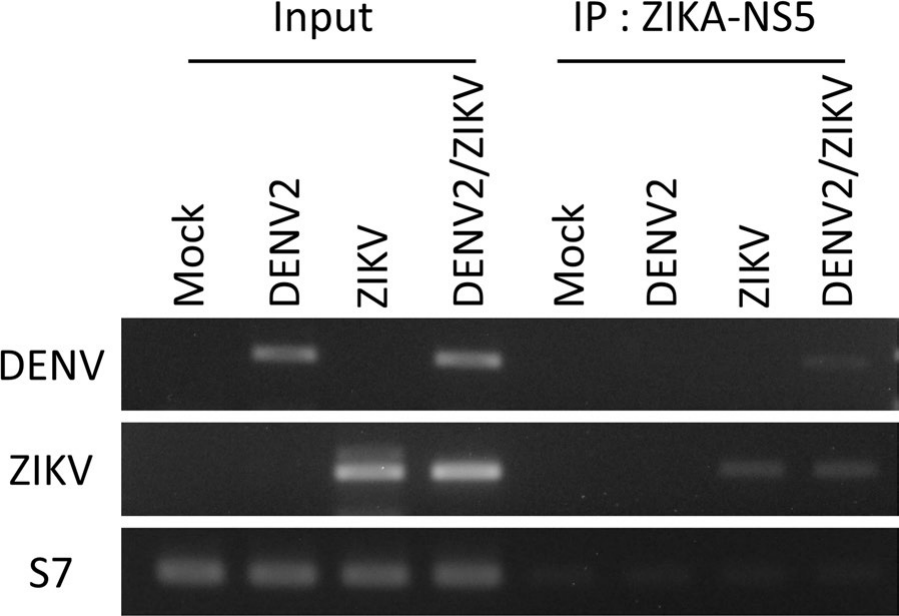
The replicating DENV2 genome interacts with the ZIKV-NS5 protein in co-infected mosquito cells. ZIKV-NS5 interacting with DENV2 and ZIKV genomic RNA was precipitated from cell lysates at 4 days using CLIP-PCR, followed by RT-PCR with primers specific for DENV and ZIKV genomes. Ribosomal protein S7 was used as a loading control

To identify specific molecular interactions between DENV2 and ZIKV in co-infected cells, we assayed each respective genome against RC-forming non-structural proteins of the complementary virus using CLIP-PCR. We identified a cognate interaction between the replicating DENV2 template single-stranded RNA (ssRNA) and ZIKV-NS5 (Fig. 3), perhaps the prominent DENV-ZIKV interaction contributing to enhanced virus replication in co-infected cells, which overall favored DENV2. This phenotype is striking, and bears important implications for *Ae. aegypti* vector competence in vivo. Apparently, DENV2 may engage the highly conserved RNA-dependent RNA polymerase ZIKV-NS5 and its RNA-capping methyltransferase activity and, perhaps, vice versa (wherein ZIKV engages DENV2-NS5) to promote its replication, amounting to greater virus production overall. Thus, to determine whether this underlying phenomenon affects vector competence in vivo, we challenged *Ae. aegypti* females with DENV2 and ZIKV in infectious blood meal.

### Co-infection of *Ae. aegypti* with DENV2 and ZIKV results in differential viral genome expression

Finding significant enhancement of viral replication by DENV2-ZIKV co-infection in vitro possibly arising from cross-species interactions, we challenged adult female *Ae. aegypti* sequentially with both viruses to quantify the effects of DENV2-ZIKV co-infection on vector competence in vivo. So far, existing studies into DENV-ZIKV co-infection have only challenged *Ae. aegypti* simultaneously, presenting both viruses in the same blood meal (BM). Contrarily, we elected to challenge mosquitos with DENV2 and ZIKV sequentially to maximize ecological validity. It is much more likely that in a DENV-ZIKV co-endemic area, a female mosquito would acquire co-infection in sequential feeding episodes: by feeding first on a DENV-infected host, then a ZIKV-infected host, or vice versa. The possibility of a mosquito obtaining both viruses from a DENV-ZIKV co-viremic human host through a single BM is much lower. Critically, we also consider the findings of studies reporting that non-infectious BM prior to subsequent viral BM can promote viral replication, as the initial non-infectious BM induces physiological changes in the midgut epithelium, rendering it more permissible to dissemination through the basal lamina [28, 29, 33, 34].

**Fig. 4:**
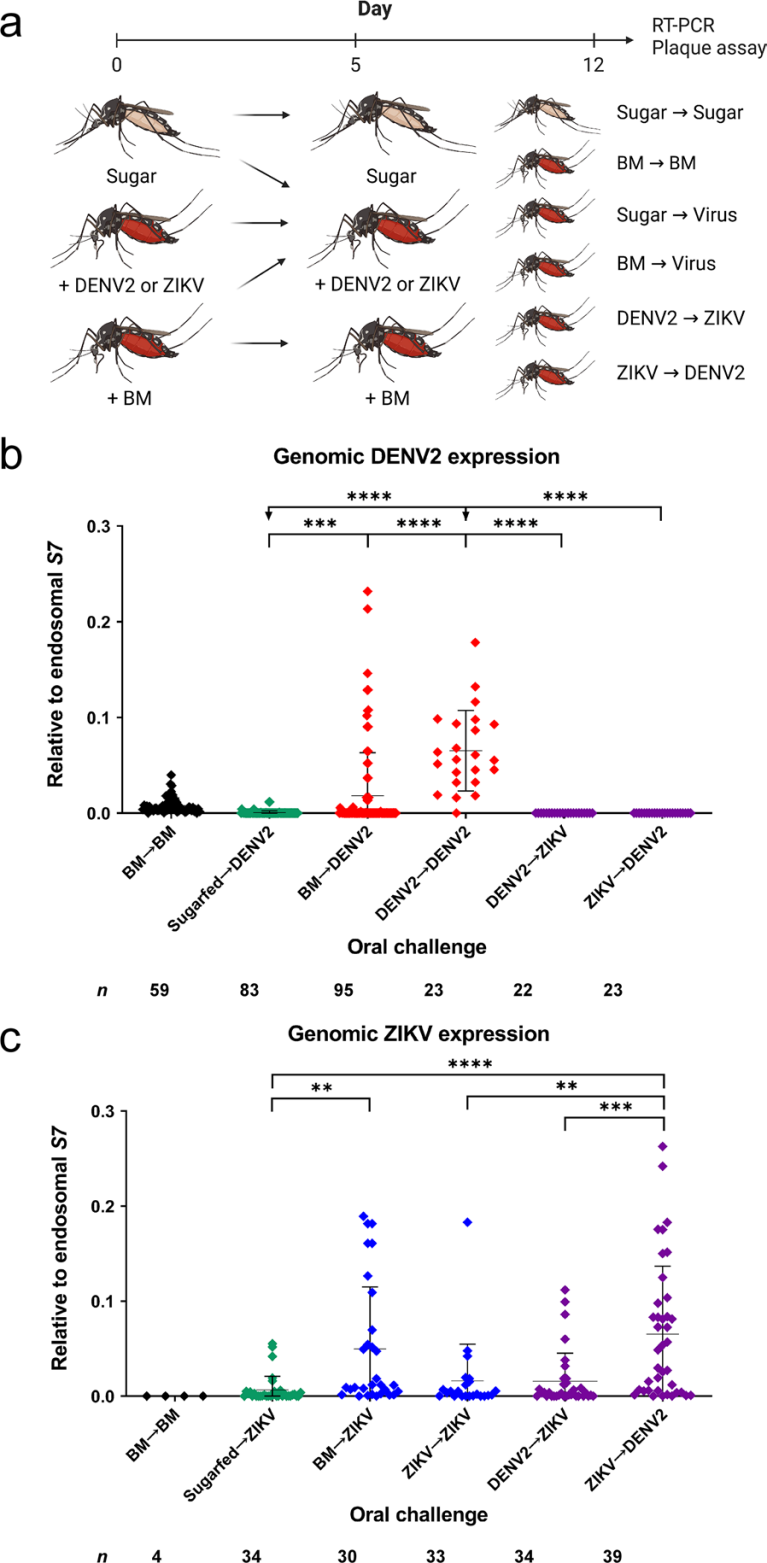
Co-infection of *Ae. aegypti* mosquitos with DENV and ZIKV results in differential viral genome expression. **A** Time course of experimental oral challenge with DENV2 and ZIKV. Mosquitos were captured 12 h ahead of day 0 and presented with blood meal (BM), DENV2, ZIKV, or maintained on sugar feeding (Sugar). On day 5, one group was given a second BM (BM-BM), and others were challenged with DENV2 or ZIKV. At 7 dpi (day 12), whole mosquito bodies were collected and homogenized. Relative viral genome expression of **B** DENV2 and **C** ZIKV was determined by qRT-PCR analysis, with normalization to the endosomal *S7* protein. Each of six oral challenge schemes consisted of at least five biologically independent cohorts, and post hoc comparisons between groups were performed using Tukey’s multiple comparisons test; **P < 0.01; ***P < 0.001; ****P < 0.0001

Thus, we included cohorts of mosquitoes presented with an initial naïve BM before challenge with DENV2 or ZIKV to account for this possible confounder in evaluating co-infection effects on viral replication (Fig. 4A). Overall, two co-infection schemes were used in vivo, in which *Ae. aegypti* were either challenged first with DENV2 and then ZIKV on a second BM (DENV2 → ZIKV), or vice versa (ZIKV → DENV2). These cohorts were compared against mosquitos single-infected with DENV and ZIKV, through either one or two BMs, as well as a mock cohort (BM-BM).

Collecting mosquitos at 7 dpi, which we previously determined to be an optimal time point for viral genomic analysis, we quantified via qRT-PCR the relative expression of DENV2 and ZIKV genomes, respectively (Fig. 4A). We found that, contrary to our observations in vitro, DENV2 expression was significantly downregulated in both co-infection scenarios, particularly when compared with those challenged twice (DENV2 → DENV2) with DENV2 (Fig. 4B). Also, a significant difference in expression between DENV2 single-infected mosquitos presented with (BM → DENV2), and without (sugar-fed → DENV2), an initial naïve BM suggests that BM-induced modifications do indeed promote viral replication by encouraging dissemination from the midgut. Genomic expression of ZIKV was significantly elevated in both co-infection scenarios, even in comparison to mosquitos infected twice (ZIKV → ZIKV) with ZIKV (Fig. 4C). Between the two co-infection groups, ZIKV → DENV2 mosquitos expressed genomic ZIKV at significantly higher levels than did their DENV2 → ZIKV counterparts. There was also a significant difference in expression between sugar-fed → ZIKV and BM → ZIKV individuals, as with DENV2, again highlighting the initial BM’s ability to promote viral replication in and past the midgut. Moving forward, we evaluated the implications of co-infection on vector competence, quantifying the infectivity of virus particles produced in vivo.

### Virus production and vector susceptibility are enhanced in *Ae. aegypti* challenged with both DENV2 and ZIKV

**Fig. 5:**
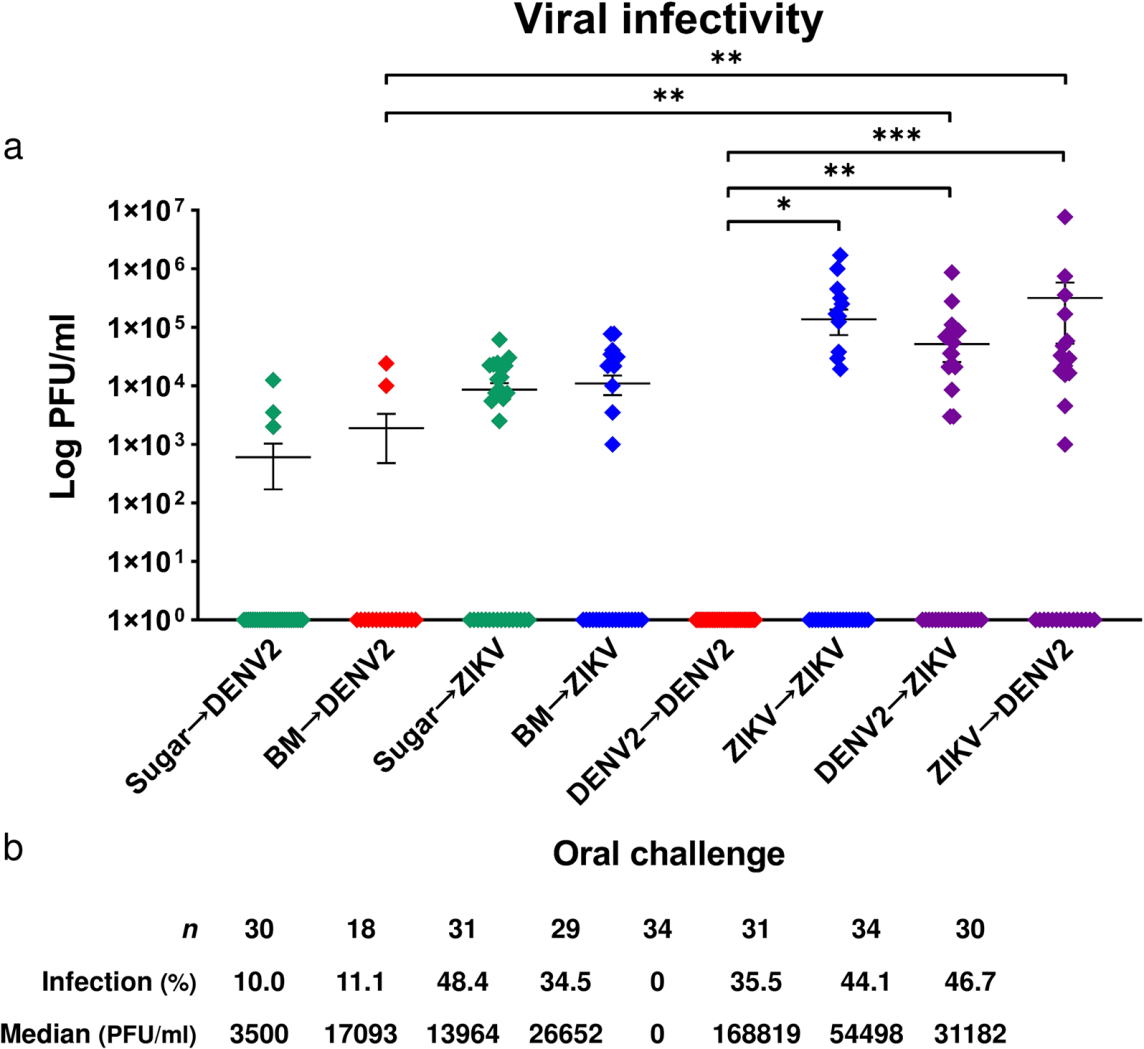
Virus production and vector susceptibility are enhanced in DENV2/ZIKV co-infected mosquitos*.* **A** Mosquitos were challenged with virus as described in Fig. 4, and then collected at 7 dpi (day 12) for plaque assay. Geometric means (PFU/ml) are plotted, and each of eight viral challenge schemes comprised at least three biological cohorts. **B** Sample size *n*, infection rate, and median PFU/ml corresponding to the experimental groups. Infected samples had positive (> 0) PFU/ml values; uninfected, negative samples are represented on the log scale as positive (PFU/ml = 1) only for visual interpretation. Comparison between groups post hoc was performed using Dunn’s multiple comparisons test; *P < 0.05; **P < 0.01; ***P < 0.001

Having characterized the genomic expression profiles of co-infected mosquitos, we next assessed the quantity and infectivity of viruses produced therein. Performing plaque assays on isolated virus from individuals collected at 7 dpi from each treatment group (Fig. 4A), we found that viral titers were significantly higher in co-infected mosquitos than in the DENV single-infected cohort (Fig. 5A). Notably, these increases were observed for both DENV2 → ZIKV and ZIKV → DENV2 co-infection groups against all three DENV single-infection cohorts: those challenged either once (sugar/BM → DENV2) or twice (DENV2 → DENV2) with DENV2. Accordingly, the corresponding infection rates were markedly higher in co-infected mosquitos, increasing by as much as 36.7% (Fig. 5B). Compared with ZIKV single-infected cohorts, viral titers were also higher in co-infected individuals. Infection rates also saw increases by about 10% except when compared with the sugar → ZIKV cohort, a result likely attributable to individual variability. Despite the unexpectedly high infection rate, the median viral titer for this cohort was at least twofold lower than those of co-infected cohorts. Overall, mosquitos challenged sequentially with DENV and ZIKV produced more infectious virus, which induced a greater extent of infection ex vivo.

Mosquitos twice-challenged with ZIKV produced higher viral titers than those co-infected, an unsurprising result following consecutive ZIKV infection. Secondary infection is greatly assisted by the existing RC infrastructure and an infected, compromised midgut epithelium and basal lamina (ZIKV → ZIKV). Indeed, viral titers were higher in co-infected mosquitos compared with those challenged once (sugar/BM → ZIKV). Infection rates of co-infected mosquitos also increased, suggesting greater susceptibility to infection and virus propagation, i.e., enhanced vector competence. As for mosquitos twice-challenged with DENV2, the clear enhancement of viral replication by co-infection is much more readily apparent, as these cohorts were entirely refractory to infection (0% infected, DENV2 → DENV2). Across DENV2 single-infected cohorts, infection rates peaked at 11.1%. The fact that vector infection rates were elevated to 44.1% and 46.7%, respectively, in co-infected cohorts indicates that DENV2-ZIKV co-infection positively, mutually modulates viral replication.

Of note, mosquitos given an initial naïve BM neither produced significantly higher viral titers nor were infected at higher rates than those directly challenged with DENV2/ZIKV. This suggests that while an initial non-infectious BM may correlate with higher viral genomic expression by assisting virus dissemination from the midgut, it ultimately neither enhances the production of viable, infectious particles nor promotes co-infected *Ae. aegypti* susceptibility to infection.

Taken together, our results show that DENV2-ZIKV co-infection significantly enhances virus production and vector susceptibility to infection. At the cellular level, viral replication is mutually enhanced owing to the overlap of highly homologous flaviviral replication machinery. We observed therein that DENV2 transcripts engage ZIKV-NS5. Overall, our findings raise grave concerns about DENV2 and ZIKV co-circulation, which threatens to strain healthcare resources and exacerbate transmission and disease as mosquitos vector these flaviviruses with greater efficiency.

## Discussion

DENV and ZIKV are flaviviruses transmitted by mosquitos of the *Aedes* genus, primarily *Ae. aegypti* [35]. They co-circulate in overlapping endemic areas. Consequently, as the spread of DENV and ZIKV expands rapidly, mosquitos will have increased opportunities to acquire simultaneous and/or mixed infections with different types of flaviviruses [35]. This may occur following an infectious BM from a single human co-viremic for DENV and ZIKV, or when mosquitos acquire sequential BMs from two individuals, each infected with a different virus. DENV and ZIKV share a highly conserved non-structural protein repository consisting of five enzymes/subunits (NS1–5), which associate closely to form a tightly-regulated RC. We hypothesized that DENV and ZIKV interact through their homologous RC components during replication, which has significant implications for viral replication and vector competence.

In the present study, we observed significant enhancements in virus production and vector susceptibility following DENV2-ZIKV co-infection in *Ae. aegypti*, and we demonstrated the cellular and molecular bases of these effects. In co-infected mosquito cells, we found that DENV2 expression was significantly enhanced, whereas ZIKV was coincidentally markedly suppressed and virus production was significantly increased for both. The finding that flaviviral RCs co-localize extensively in the cytoplasm, we determined whether DENV and ZIKV participate in cross-species interactions during replication. We found that the replicating DENV2 genome engages ZIKV-NS5. This surprising interaction readily explains the drastic enhancement of DENV2 expression in vitro, which suggests that DENV2 engages the ZIKV RC competitively. DENV-ZIKV interactions during replication provide a basis for the mutual increase in secretory virus particles observed. Next, we challenged *Ae. aegypti* adult females with both viruses to determine whether similar DENV2-ZIKV interactions also modulate viral replication in vivo. We found that while genomic expression at 7 dpi of DENV2 was markedly downregulated, with ZIKV upregulated, there was again a mutual enhancement of viral replication as indicated by significantly elevated levels of infectious virions produced in co-infected mosquitos, which were also more susceptible to infection.

Our findings describe for the first time the potent mutualistic outcomes of flaviviral co-infection for viral replication, and suggest that vector competence may be enhanced as a result. Vector competence in the arboviral sylvatic cycle is defined by the extent to which the mosquito permits a virus to utilize its circulatory system. The virus must first establish infection in the midgut and produce virions that disseminate to secondary tissues, which must then sustain replication throughout the organ system until freshly propagated virions breach the salivary glands. We found that viruses produced in DENV-ZIKV co-challenged mosquitos were significantly more abundant compared with those infected with only one virus, particularly DENV2. Moreover, these mosquitos were infected at a higher rate, suggesting that co-infected mosquitos are more susceptible to infection, perhaps resulting from the convergence of immune suppression pathways of both replicating viruses, particularly in the midgut. These results strongly suggest that co-infection enhances vector competence because, at a collection date of 7 dpi, either virus will have already disseminated from the midgut and commenced replication throughout the entire body to produce viable, infectious particles. Although employing more collection points to visualize replication kinetics may allow for a clearer spatio-temporal resolution, for the purpose of understanding co-infection in terms of viral replication and vector competence, it was sufficient to isolate virus from mosquitos at 7 dpi for the plaque assay. The implications of our results for vector competence are somewhat limited, however, absent organ-specific analysis. Specifically, quantifying virus present in the salivary glands may permit more direct observation of outcomes for vector competence, as infectious viral particles must be secreted into the saliva during a BM for transmission [56]. However, the variability of flaviviral infection in vivo among individual mosquitos largely precluded such an investigation, as salivary glands dissected and analyzed individually would likely vary greatly in viral titer. Collecting whole bodies enabled an individual analysis without sacrificing statistical integrity. Additionally, we studied replication dynamics in vivo using a blood meal challenge, opting not to infect mosquitos by intrathoracic injection to preserve the critical barrier to vector competence manifest in the midgut’s physical and immunological fortifications. Quantifying virus propagated through sequential co-infection by oral challenge allowed us to observe the consequences of DENV-ZIKV interaction directly through the entire course of infection within the vector, beginning with ingestion in the midgut. Furthermore, our findings are highly relevant to the evolving landscape of DENV-ZIKV endemicity, as our sequential oral infection model is closely aligned with actual vector activity. Mosquitos are much more likely to acquire flaviviral co-infection sequentially from individual hosts than from a single host viremic for both DENV and ZIKV. Individual variability in feeding opportunity, i.e., extent of engorgement, however, likely negates many real differences in virus intake between laboratory and field.

To date, exploratory studies into arboviral co-infection have only established that *Ae. aegypti* are susceptible to infection by more than one type of virus and they may simultaneously transmit multiple viruses. One study reported that *Ae. aegypti* simultaneously challenged with a combination of two or three arboviruses including DENV2, ZIKV, and chikungunya (CHIKV), were frequently double- or triple-infected, which indicated that the mosquitos are susceptible to co-infection. Inoculation of saliva in vitro confirmed the potential for co-transmission of all three viruses [12]. Another study found that mosquitos simultaneously co-infected with DENV and ZIKV preferably transmit the latter [24]. The co-infection and co-transmission potential of ZIKV-CHIKV [36, 37] and DENV-CHIKV [38] have also been supported. With respect to arboviral co-infection*,* our study contributes novel insight into its implications for viral propagation in *Ae. Aegypti* and demonstrates that DENV-ZIKV co-infection mutually enhances viral replication. Our results also suggest that vector competence may be enhanced following DENV-ZIKV co-infection, as indicated by increased infection rates. We also provide the first account of molecular mechanisms underlying co-infection effects by reporting that DENV engages ZIKV-NS5 during replication.

As for the apparent conflict between our in vitro and in vivo results in which DENV2 replication appeared be competitively promoted in co-infected cells (Figs. 1B, 2C), but drastically suppressed in the mosquito (Fig. 4), it is important to note that markedly reduced DENV2 expression in co-infected mosquitos reflected only the viral genome content at 7 dpi, not the amount of infectious DENV virions produced by the vector. Indeed, virus production was significantly higher in co-infected mosquitos than in those single-infected with DENV. In addition, co-infected cohorts were much more susceptible to infection. Low DENV2 genomic expression suggests that there may be competitive engagement between ZIKV and DENV2 due to interaction between ZIKV genomic transcripts with DENV2 RC proteins. We showed that DENV2 utilizes ZIKV-NS5 for transcription; thus, it is likely that ZIKV may reciprocally utilize DENV2-NS5 to its advantage. Another intriguing possibility is that flaviviral NS5 may have a dual purpose in the convergence of DENV-ZIKV co-infection by cross-species capping of the respective RNA genomes by N-terminus methyltransferases to assist in evasion of the host immune response. This is an alternative (and not mutually exclusive) molecular premise for the observed increases in virus production and vector susceptibility in co-infected mosquitos. Further biochemical studies regarding the DENV-ZIKV interaction via NS5, a possible target for vector control and vaccine development, is warranted as the co-circulation of DENV and ZIKV broadens globally.

The clinical and epidemiological implications of expanding flaviviral co-circulation remain largely unexplored. Though it is not clear whether co-infected patients develop more severe disease [39–42], increased co-circulation and transmission of multiple flaviviruses will surely pose significant problems for diagnosis and surveillance because of the common clinical presentation, asymptomatic response, and cross-reactivity. Although progress is being made on the diagnostics front [43–45], effective vector control remains the most effective approach to managing and eliminating mosquito-borne diseases [3]. Central to vector control is a clear understanding of the pathogen-vector relationship as it evolves in real-time, with expanding arboviral co-circulation being a worrying trend that we have addressed. As the periphery of DENV-ZIKV co-circulation expands, it also encompasses other arboviruses, such as CHIKV [46]. As we demonstrated, co-infection with DENV and ZIKV mutually enhances viral replication within the vector. Further interaction with other arbovirus, such as yellow fever virus or CHIKV, may result in unknown synergistic effects. This may similarly threaten to facilitate widespread circulation and transmission of multiple deadly viruses by modulating the vector response. The interplay among these arboviruses in *Ae. aegypti* and its effects on viral replication and vector competence require further study. Future work in this area could incorporate the study of viral interactions with the mosquito microbiota [47, 48] and diverse RNA responses [49–52] of the mosquito. Whether differential interactions arise between the DENV serotypes in co-infection scenarios is also worth pursuing, as all four DENV serotypes (DENV1–4) are spreading throughout Asia, Africa, and the Americas [53]. It also remains to be determined whether arbovirus co-infection influences virus selection pressure and recombination events [54, 55]. In conclusion, this study presents the novel finding that DENV-ZIKV co-infection mutually enhances viral replication within the mosquito. This threatens to increase the disease burden in co-endemic areas, drive the “silent” transmission of strains not predominantly circulating, and introduce flaviviruses into communities not yet seen. With arboviral co-endemicity on the rise globally, the rapidly shifting vector–pathogen relationship must be further investigated, in which the pathogen itself bears many faces.

## Conclusions

In this study, we determined the effects of DENV and ZIKV co-infection on viral replication in *Ae. aegypti* and to identify the specific molecular interactions involved. We first examined viral replication dynamics in cells infected simultaneously or sequentially with DENV and ZIKV. We report the interspecies binding of viral genomic transcripts to NS5. We then challenged *Ae. aegypti* mosquitos with both DENV2 and ZIKV sequentially to identify similar interactions, and found that virus production and vector susceptibility to infection were significantly enhanced. Our results suggest that DENV2 and ZIKV simultaneously establish infection in the *Ae. aegypti* vector, which may mutually augment one another during replication. The data also implicate the homologous NS5 protein as a key intersection between the flaviviruses in co-infection, highlighting it as a potential target for vector control.

## Supplementary Information

**Additional file 1:** **Figure S1.** DENV2 and ZIKV single-infected cells are phenotypically distinct. **A** Cells inoculated with either DENV2 or ZIKV at MOI = 1 were stained for DAPIand flaviviral NS1at 2 dpi. **B** Cell density imaged under bright field revealed that DENV2-infected cells were less numerous than ZIKV-infected cells. Representative images shown.

## Data Availability

All data generated or analyzed during this study are included in this published article.

## Abbreviations

BM: Blood meal
BSA: Bovine serum albumin
dpi: Days post-infection
DENV: Dengue virus
ICR: Institute of Cancer Research
IFA: Immunofluorescence assay
MOI: Multiplicity of infection
PE: Post-eclosion
RCs: Replication complexes
ZIKV: Zika virus
