# Supplementary figures and images for: Co-infection of dengue and Zika viruses mutually enhances viral replication in the mosquito Aedes aegypti
Source: Parasit Vectors. 2023 May 11;16:160. doi: 10.1186/s13071-023-05778-1 (PMC10172068; doi:10.1186/s13071-023-05778-1)

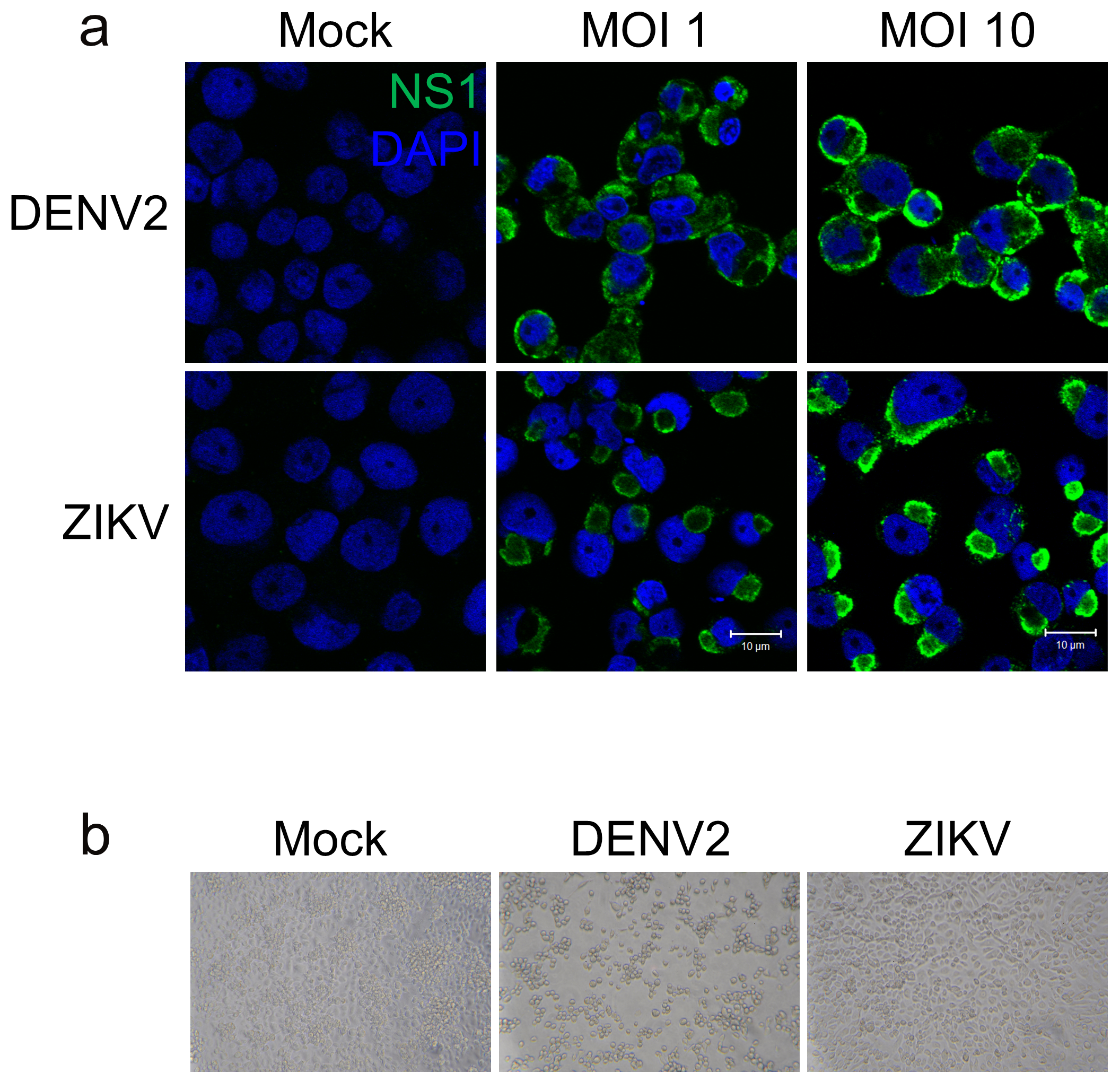

Supplement: Supplementary file 1 — Additional file 1: Figure S1. DENV2 and ZIKV single-infected cells are phenotypically distinct. A Cells inoculated with either DENV2 or ZIKV at MOI = 1 were stained for DAPIand flaviviral NS1at 2 dpi. B Cell density imaged under bright field revealed that DENV2-infected cells were less numerous than ZIKV-infected cells. Representative images shown. [file 13071_2023_5778_MOESM1_ESM.tif]
